# A Fusion Algorithm for GFP Image and Phase Contrast Image of Arabidopsis Cell Based on SFL-Contourlet Transform

**DOI:** 10.1155/2013/635040

**Published:** 2013-02-14

**Authors:** Peng Feng, Jing Wang, Biao Wei, Deling Mi

**Affiliations:** Key Laboratory of Optoelectronics Technology & System, Ministry of Education, Chongqing University, Chongqing 400044, China

## Abstract

A hybrid multiscale and multilevel image fusion algorithm for green fluorescent protein (GFP) image and phase contrast image of Arabidopsis cell is proposed in this paper. Combining intensity-hue-saturation (IHS) transform and sharp frequency localization Contourlet transform (SFL-CT), this algorithm uses different fusion strategies for different detailed subbands, which include neighborhood consistency measurement (NCM) that can adaptively find balance between color background and gray structure. Also two kinds of neighborhood classes based on empirical model are taken into consideration. Visual information fidelity (VIF) as an objective criterion is introduced to evaluate the fusion image. The experimental results of 117 groups of Arabidopsis cell image from John Innes Center show that the new algorithm cannot only make the details of original images well preserved but also improve the visibility of the fusion image, which shows the superiority of the novel method to traditional ones.

## 1. Introduction

**Figure 1:**
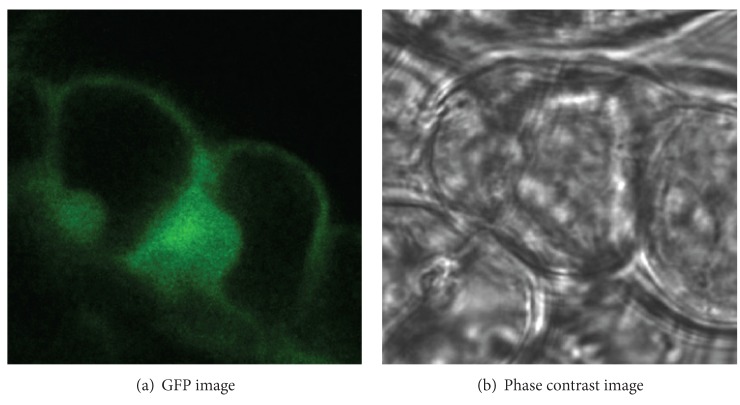
Arabidopsis cell images.

The purpose of image fusion is to integrate complementary and redundant information from multiple images of the same scene to create a single composite that contains all the important features of the original images [1]. The resulting fused image will thus be more suitable for human and machine perception or for further image processing tasks in many fields, such as remote sensing, disease diagnosis, and biomedical research. In molecular biology, the fluorescence imaging and the phase contrast imaging are two common imaging systems [2]. Green fluorescent protein (GFP) imaging can provide the function information related to the molecular distribution in biological living cells; phase contrast imaging provides the structural information with high resolution by transforming the phase difference which is hardly observed into amplitude difference. The combination of GFP image and phase contrast image is valuable for function analyses of protein and accurate localization of subcellular structure.  Figure 1 shows one group of registered GFP image and phase contrast image for Arabidopsis cell; it is obvious that there is a big difference between the GFP image and the phase contrast image. Due to low similarity between the originals, various fusion methods that had been widely used in remote image fusion [3–5], such as Wavelet/Contourlet-based ARSIS fusion method [6], will result in spectral and color distortion, dark and nonuniform background, and poor ability of detailed preservation. Recently, Li and Wang have proposed SWT-based (stationary wavelet transform) [7] and NSCT-based (nonsubsampled Contourlet transform) [8] fusion algorithms which utilize the translation invariance of two kinds of transform to reduce the artifacts of fused image, but complicated procedure, high time-consumption, and low robustness hinder its fusion capability. In order to overcome these disadvantages, we bring sharp frequency localization Contourlet transform (SFL-CT) [9] into the fusion of GFP image and phase contrast image, in the manner of SFL-CT's merit of excellent edge expression ability, multiscale, directional characteristics, and anisotropy. We propose a new hybrid multiscale, and multilevel image fusion method combining intensity-hue-saturation (IHS) transform and SFL-CT. Different fusion strategies are utilized for the coefficients of different subbands in order to keep the localization information in GFP image and detailed information of high resolution in phase contrast image. The research conducts a fusion test of 117 groups of Arabidopsis cell images from the GFP database of John Innes Center [10]. Visual information fidelity (VIF) [11] is also introduced to quantify the similarity inside and outside the fluorescent area between the fused image and original ones.

The outline of this paper is as follows. In Section 2, the SFL-CT and IHS transforms are introduced in detail.  Section 3 concretely describes our proposed fusion algorithm based on the neighborhood consistency measurement. Experimental results and performance analysis are presented and discussed in Section 4.  Section 5 gives the conclusion of this paper.

## 2. SFL-Contourlet Transform and IHS Transform

### 2.1. Traditional Contourlet Transform

In 2005, Do and Vetterli [12] proposed the Contourlet transform as a directional multiresolution image representation that can efficiently capture and represent smooth object boundaries in natural images. The Contourlet transform is constructed as a combination of the Laplacian pyramid transform (LPT) [13] and the directional filter banks (DFB) [14], where the LPT iteratively decomposes a 2D image into low-pass and high-pass subbands, and the DFB are applied to the high-pass subbands to further decompose the frequency spectrum into directional subbands.

**Figure 2:**
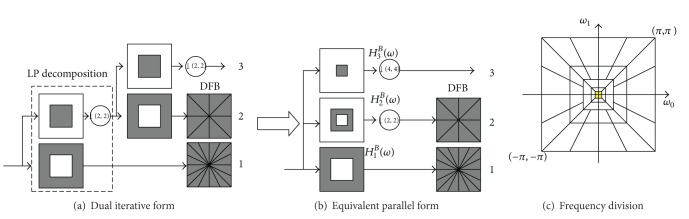
Block diagram of Contourlet transform with 2 levels of multiscale decomposition.

The block diagram of the Contourlet transform with two levels of multiscale decomposition is shown in Figure 2(a), followed by angular decomposition. Note that the Laplacian pyramid shown in the diagram is a simplified version of its actual implementation. Nevertheless, this simplification serves our explanation purposes satisfactorily. By using the multirate identities, we can rewrite the filter bank into its equivalent parallel form, as shown in Figure 2(b), where *H*_*i*_^*B*^(*ω*), *i* = 1,2, 3, is the equivalent filter of LPT for each decomposition level [15]. Obviously, using ideal filters, the Contourlet transform will decompose the 2D frequency spectrum into trapezoid-shaped regions as shown in Figure 2(c).

**Figure 3:**
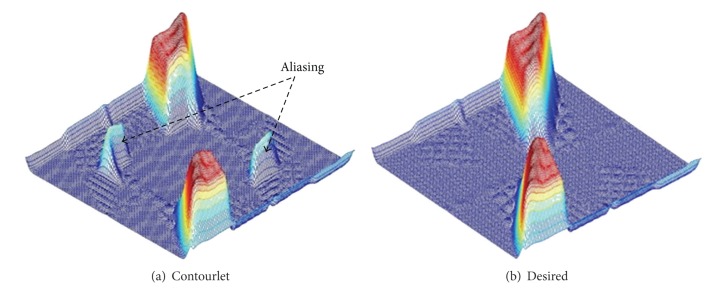
Frequency support of one channel for Contourlet transform and desired scheme.

Due to the periodicity of 2D frequency spectrums for discrete signals and intrinsic paradox between critical sample and perfect reconstruction of DFB, it means that we cannot get perfect reconstruction and frequency domain localization simultaneously by a critically sampled filter bank with the frequency partitioning of the DFB. When the DFB is combined with a multiscale decomposition as in the Contourlet transform, the aliasing problem becomes a serious issue. For instance, Figure 3(a) shows the frequency support of an equivalent directional filter of the second channel in Figure 2(b). We can see that Contourlets are not localized in the frequency domain, with substantial amount of aliasing components outside of the desired trapezoid-shaped support as shown in Figure 3(b).

### 2.2. Sharp Frequency Localization Contourlet Transform

**Figure 4:**
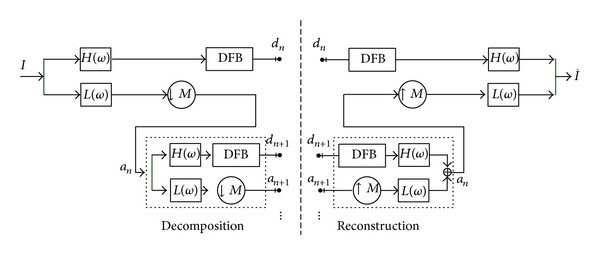
Block diagram of SFL-CT.

In order to overcome the aliasing disadvantage of Contourlet transform, Lu proposed a new construction scheme which employed a new pyramidal structure for the multiscale decomposition as the replacement of LPT [15]. This new construction is named as sharp frequency localization Contourlet transform (SFL-CT) [9], and its block diagram is shown in Figure 4.

In the diagram, *H*(**ω**) represents the high-pass filter, and *L*(**ω**) represents low-pass filter in the multiscale decomposition, with **ω** = (*ω*_0_, *ω*_1_). The DFB which is the same as in Contourlet transform (CT) is attached to the high-pass channel at the finest scale and bandpass channel at all coarser scales. The low-pass filter *L*(**ω**) in each levels is downsampled by matrix *M*, with *M* normally being set as diagonal matrix (2,2). To have more levels of decomposition, we can iteratively insert at point *a*_*n*+1_ a copy of the diagram contents enclosed by the dashed rectangle. As an important difference from the LPT shown in Figure 2, the new multiscale pyramid can employ a different set of low-pass and high-pass filters for the first level and all other levels, and this is a crucial step in reducing the frequency-domain aliasing of traditional Contourlet transform. We leave the detailed explanation for this issue as well as the specification of the filters *H*(**ω**) and *L*(**ω**) to [9].

**Figure 5:**
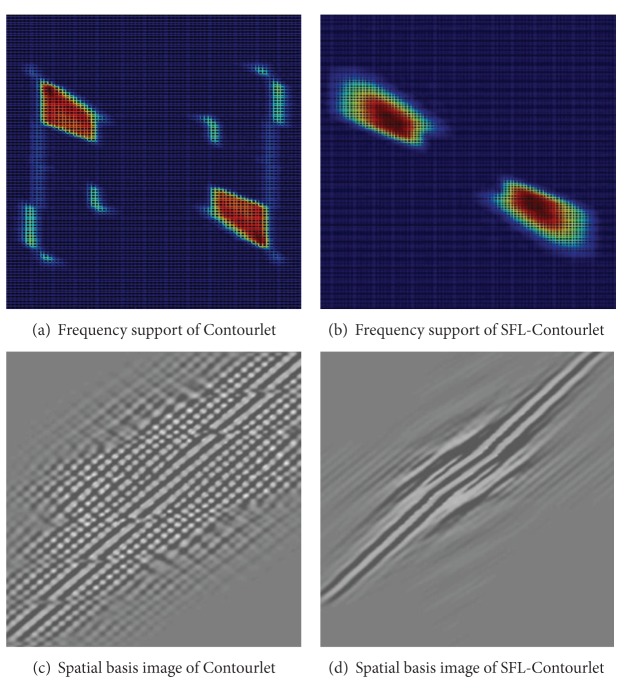
Comparison of basis image.

Figure 5 shows one Contourlet basis image and its corresponding SFL-Contourlet part in the frequency and spatial domains. As we can see from Figure 5(a), the original Contourlet transform suffers from the frequency nonlocalization problem. In sharp contrast, SFL-Contourlet produces basis image that is well localized in the frequency domain, as shown in Figure 5(b). The improvement in the frequency localization is also reflected in the spatial domain. As shown in Figures 5(c) and 5(d), the spatial regularity of SFL-Contourlet is obviously superior to the one of Contourlet. 

### 2.3. IHS Transform

The intensity-hue-saturation (IHS) transform substitutes the gray image for the intensity component of the color image and thus handles the fusion of the gray and color images [1] and defines three color attribute based on the human visual mechanism, that is, intensity (*I*), hue (*H*), and saturation (*S*). *I* stands for the information of the source image, *H* stands for the spectrum and color attributes, and *S* stands for the purity relative to the grayscale of some color. In IHS space, *H* component and *S* component are closely tied to the way that people feel about color, while *I* component almost has nothing to do with the color component of the image.

There are various algorithms that can transform image from RGB to IHS space, common transformation model including sphere transformation, cylinder transformation, triangle transform, and single six cones [16]. We use triangle transform here. The formula of the forward and inverse transforms are as follows.

From RGB to IHS space (forward transform),

(1) $$\begin{array}{c} I=\frac{R+G+B}{3},\\ S=1-\frac{3}{R+G+B}\left[\min\left(R,G,B\right)\right],\\ H=\{\begin{array}{cc} 0 & B\leq G\\ 2\pi -\alpha & B>G, \end{array} \end{array}$$

where

(2) $$\begin{array}{c} \alpha =\text{arccos}\left\{\frac{\left(1/2\right)\left[\left(R-G\right)+\left(R-B\right)\right]}{{\left[{\left(R-G\right)}^{2}+(R-B)(G-B)\right]}^{1/2}}\right\}. \end{array}$$

The reverse transform

(3)  for  0≤H<2π3,  {B=I(1−S)R=I×[1+Scos⁡(H)cos⁡⁡(π/3−H)]G=3I−(R+B),for  2π3≤H<4π3,  {B=I(1−S)R=I×[1+Scos⁡(H−2π/3)cos⁡⁡(π/3−H)]G=3I−(R+B),for  4π3≤H<2π,  {B=I(1−S)R=I×[1+Scos⁡(H−4π/3)cos⁡⁡(π−H)]G=3I−(R+B).

## 3. The Proposed Fusion Rule

**Figure 6:**
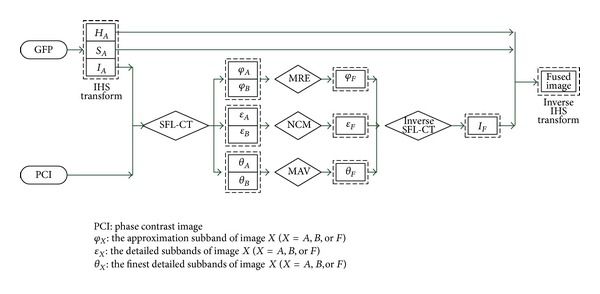
Schematic diagram of the proposed image fusion algorithm, where subscripts *A*, *B*, and *F* mean GFP image, phase contrast image, and the fused image, respectively.

From Figure 1(a), we can see that the background of the GFP image is partially dark; in order to avoid the influence of low contrast after fusion, the intensity component of the original GFP image is extracted by IHS transform which not only keeps most of the information from the original one, but also entirely improves the brightness of the fused image. In this way, we can explore a hybrid multiscale and multilevel fusion algorithm for biological cell image. We use SFL-CT to decompose the intensity components of GFP image and phase contrast image; different fusion schemes are used for different subband coefficients in order to keep a balance between the localization information in GFP image and detailed information of high frequency in phase contrast image. To get the protein distribution information of GFP image, the approximation (coarsest) subband coefficients of fused image are obtained with maximum region energy rule (MRE) [17]. To get structural information of the phase contrast image, coefficients of the finest detailed subband of fused image are based on maximum absolute value rule (MAV) [17]. To balance structural information and color molecular distribution information from the originals, a locally adaptive coefficient fusion rule named neighborhood consistency measurement (NCM) is adopted on coefficients of other detailed subbands. The schematic diagram is shown in Figure 6.

### 3.1. Maximum Region Energy (MRE) Rule

When GFP image and phase contrast image are decomposed by the SFL-CT, the coefficients of the coarsest subband represent the approximation component of the input images. Considering approximate information of fused image is constructed by the two kinds of approximation subband coefficients; maximum region energy rule (MRE) is a good choice for the fused approximation subband coefficients.

MRE rule is defined as follows:

(4) cJF(m,n)={cJA(m,n),  if  EJA(m,n)>EJB(m,n)cJB(m,n),  if  EJA(m,n)≤EJB(m,n),

where the regional energy *E* is defined as

(5) $$\begin{array}{c} E_{J}^{X}\left(m,n\right)=\sum_{\left(x,y\right)\in \Omega \left(m,n\right)}{\left[c_{J}^{X}\left(x,y\right)-\mu_{J}^{X}\left(m,n\right)\right]}^{2},\\ X=A,B\;\text{or}\;F, \end{array}$$

where *E*_*J*_^*A*^(*m*, *n*),  *E*_*J*_^*B*^(*m*, *n*), and  *E*_*J*_^*F*^(*m*, *n*) denote regional energy of original image *A*, *B*, and fused image *F* in the coarsest scale *J* and location (*m*, *n*). *Ω*(*m*, *n*) represents a square region with 3 × 3 size whose center is located at position (*m*, *n*). *c*_*J*_^*X*^(*m*, *n*) denotes the coefficient of the images *X* = *A*, *B*, or *F* within the region *Ω*(*m*, *n*) in the coarsest subband *J* and location (*m*, *n*). *μ*_*J*_^*X*^(*m*, *n*) means the average value of coefficients within *Ω*(*m*, *n*).

### 3.2. Maximum Absolute Value (MAV) Rule

After decomposing the input images using SFL-CT, the image details are contained in the directional subbands in SFL-CT domain. The directional subband coefficients with larger absolute values, especially for subband coefficients at the finest scale, generally correspond to pixels with sharper brightness in the image and thus to the salient features such as edges, lines, and regions boundaries. Therefore, we can use the maximum absolute value (MAV) scheme to make a decision on the selection of coefficients at the finest detailed subbands.

MAV fusion rule is defined as follows:

(6) dj,lF(m,n)={dj,lA,  if  abs[dj,lA(m,n)]>abs[dj,lB(m,n)]dj,lB,  if  abs[dj,lA(m,n)]<abs[dj,lB(m,n)],

where *d*_*j*,*l*_^*A*^(*m*, *n*),  *d*_*j*,*l*_^*B*^(*m*, *n*), and *d*_*j*,*l*_^*F*^(*m*, *n*) denote the coefficients of the images *A*, *B*, and the fused image *F* in the *j*th scale,*l*th directional subband, and location (*m*, *n*). abs[·] denotes absolute operator. 

### 3.3. Neighborhood Consistency Measurement (NCM)

Let *N*_*j*,*l*_^*X*^(*m*, *n*) denote a region centered at coefficient *d*_*j*,*l*_^*X*^(*m*, *n*) in *j*th level and *l*th directional subband of image *X*, and the energy of this region is defined as *ρ*_*j*,*l*_^*X*^(*m*, *n*). Then,

(7) $$\begin{array}{c} \rho_{j,l}^{X}\left(m,n\right)=\sum_{\left(k,p\right)\in N_{j,l}^{X}\left(m,n\right)}{\left[d_{j,l}^{X}\left(k,p\right)\right]}^{2},\;X=A,B\;\text{or}\;F. \end{array}$$

The NCM is defined as a threshold for directional coefficients based on one region mentioned above. Let Ψ_*j*,*l*_(*m*, *n*) denote NCM as follows:

(8) $$\begin{array}{c} \Psi_{j,l}\left(m,n\right)=\frac{2\times \left\{\sum_{\left(k,p)\in N_{j,l}^{A}(m,n\right)}\left[d_{j,l}^{A}\left(k,p\right)\right]\times \left[d_{j,l}^{B}\left(k,p\right)\right]\right\}}{\rho_{j,l}^{A}\left(m,n\right)+\rho_{j,l}^{B}\left(m,n\right)}. \end{array}$$

It is not hard to see that the NCM is smaller than 1. In fact, NCM indicates whether the neighborhood is homogenous. Bigger NCM means being more homogenous.

**Figure 7:**
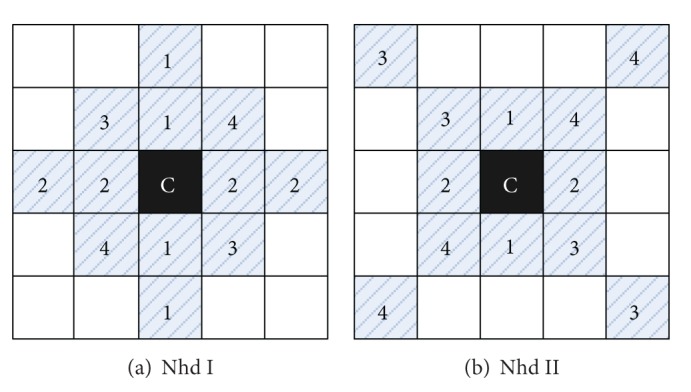
Neighborhood coefficients of SFL-CT.

**Figure 8:**
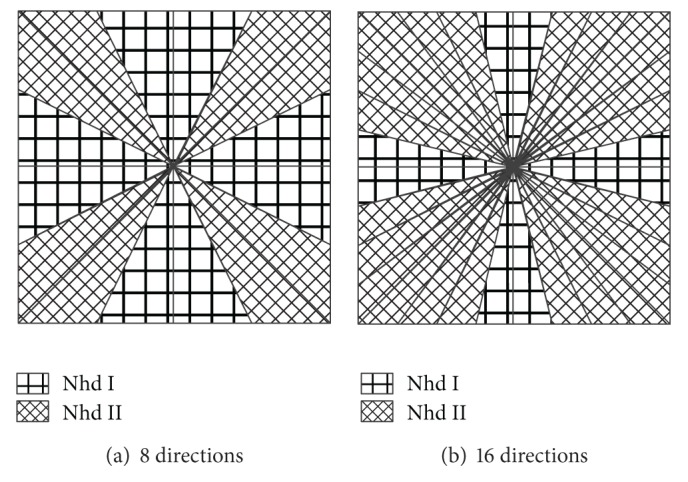
Empirical distribution model for neighborhood selection.

Taking the number of directions in each detailed subband into consideration, we classify neighborhood into two classes: Nhd I and Nhd II which are shown in Figures 7(a) and 7(b). Nhd I is mainly used in horizontal and vertical subbands, and Nhd II is in other subbands. For instance, if the direction number is 8 or 16, we can use empirical distribution model as Figure 8.

We define a threshold *T* which is normally 0.5 < *T* < 1.  

If Ψ_*j*,*l*_(*m*, *n*) < *T*, then

(9) dj,lF(m,n)={dj,lA,  if  ρj,lA(m,n)≥ρj,lB(m,n)dj,lB,  if  ρj,lA(m,n)<ρj,lB(m,n).

If Ψ_*j*,*l*_(*m*, *n*) ≥ *T*, then

(10) dj,lF(m,n)=Ψj,l(m,n)×max⁡⁡[dj,lA(m,n),dj,lB(m,n)]+[1−Ψj,l(m,n)]×min⁡⁡[dj,lA(m,n),dj,lB(m,n)].

### 3.4. Fusion Procedure

The fusion process, accompanied with the proposed fusion rule, is carried out as in the following steps.

1. Define the register original images: GFP image as image *A*, phase contrast image as image *B*, and fused image as image *F*.
2. Make IHS transform for image *A*, and calculate the corresponding intensity components *I*_*A*_, hue component *H*_*A*_, and saturation component *S*_*A*_.
3. Decompose *I*_*A*_ and image *B* by SFL-CT, and get two approximation subbands {*φ*_*A*_, *φ*_*B*_}, a series of the finest detailed subbands {*θ*_*A*_, *θ*_*B*_}, and other detailed subbands {*ε*_*A*_, *ε*_*B*_}.
4. Combine transform coefficients according to the selection rule: coefficients of approximation subbands are based on MRE rule; coefficients of the finest detailed subbands are based on MAV rule; coefficients of other detailed subbands are based on NCM. We can get the approximation subband *φ*_*F*_, detailed subbands *ε*_*F*_, and *θ*_*F*_ of the fused image *F*.
5. Reconstruct the intensity of the fused image *I*_*F*_ with *φ*_*F*_, *ε*_*F*_, and *θ*_*F*_ by the inverse SFL-CT.
6. Reconstruct the fused image *F* with the hue *H*_*A*_ and saturation *S*_*A*_, together with the *I*_*F*_ by the inverse IHS transform.

## 4. Experimental Results and Discussion

### 4.1. Dataset

All images used in this experiment come from the GFP database of John Innes Center [10]. The original size of images is 358 × 358 pixels. We resize and crop them into 256 × 256 pixels in order to facilitate processing. The experiment contains 117 sets of GFP images (24-bit true color) and their corresponding phase contrast images (8-bit grey scale) of the Arabidopsis. The former reveal the distribution of the labeled protein, and the latter present cell structures information.

### 4.2. Parameters Selection

For the proposed method, the practical windows (Ω) in NCM rule are usually chosen to be of size 3 × 3, 5 × 5, or 7 × 7. We have investigated these practical windows and found that size 5 × 5 provides good results considering fusion clarity and time consumption. Apart from the sizes of the practical windows, the frequency parameters of SFL-CT are also needed to choose for improving the fusion performance. A larger number of experimental results demonstrate that the passband frequency *ω*_*p*_ and stopband frequency *ω*_*s*_ which should be 4*π*/21 and 10*π*/21, respectively, can not only provide pleasing fusion performance in most cases, but also keep good balance between fusion result and computation complexity.

### 4.3. Results Comparison

We compare the proposed fusion rule with the traditional methods or rules. They include traditional IHS fusion method (T-IHS) [18], MRE and MAV fusion method based on IHS space (IHS + MRE and MAV) [1], and PCNN-based fusion method [19] in which all the images are decomposed by the nonsubsampled Contourlet transform (NSCT + PCNN), and our method (Hybrid NCM). Among them, MAV stands for the maximum absolute value rules; MRE stands for maximum region energy rules; MRE and MAV represents MRE rule for approximation subband and MAV rule for detailed subbands. The parameters of the above method are set as follows. For the rule of the fusion of MRE, neighborhood window is of size 3 × 3 pixels. SFL-CT makes a decomposition for 4 layers; the numbers of directions of each layer are (4, 8, 16, and 16); the filter for DFB is “pkva” filter; the set of NSCT + PCNN fusion algorithm is just the same as that in [20].

**Figure 9:**
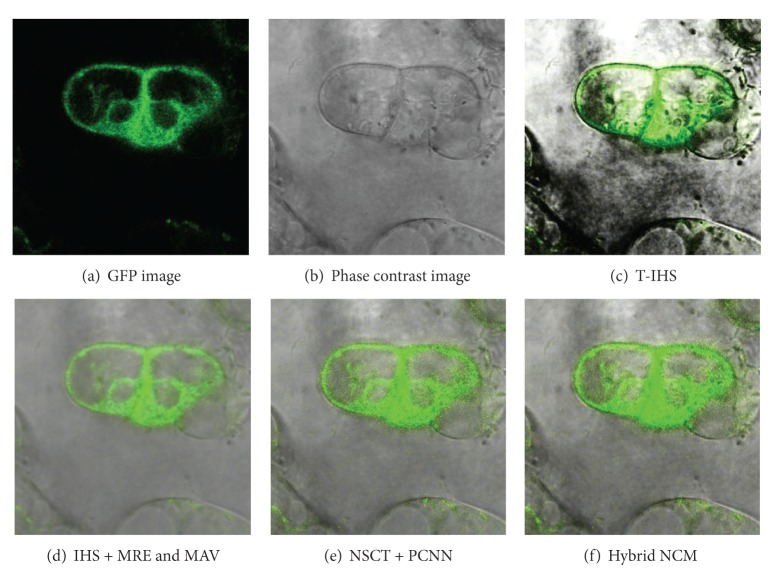
Fused images using different methods.

The fusion results, shown in Figure 9, which are obtained by four different methods demonstrate visual difference. It is obvious to see that the fused image using T-IHS method is unsatisfactory. The foreground and background are significantly nonuniform, especially along the cell outlines as there exist fuzzy blacks, so it is difficult to distinguish the inner information. However, the brilliance shown in Figures 9(d)–9(f) is largely improved, and the details of the images are also clearer. All in all, the location information of the cell structure in the phase contrast image and the distribution information of the protein are largely retained. Nevertheless, it is not easy to objectively judge the quality of the above three methods. For better judging these fusion results, the quantitative parameter that is visual information fidelity (VIF) [11] is taken into consideration. In the recent studies, large-scale subjective experiments assess VIF, a novel image similarity criterion, and prove it to be a good substitution for the subjective assessment. We know that there are two kinds of traditional evaluations that are subjective evaluation and objective evaluation. The former depends on the perception of human eye vision; different people would have different perception. The latter method has a little link with subjective factor, but it does not well measure the difference between the fusion image and the original image. As for the characteristic, that is, the little similarity between GFP fluorescence image and phase image, the VIF method, which is the combination of human visual system (HVS) and image characteristic statistics, is introduced into this paper to measure the quality of fusion image. This method can tell us the similarity between different regions of fusion image and the original image in quantitative aspect. The VIF value (the range is 0~1) is closer to 1; then it indicates that the fusion image has more similarity to the original image. A number of experimental results have proved that the VIF method and the human subjective evaluation have a better similarity for image quality than the traditional methods such as root mean square error (RMSE), correlation coefficients (CCs), and mutual information (MI).

**Figure 10:**
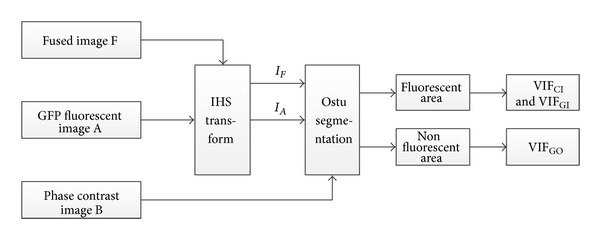
VIF algorithm flow chart.

Considering the difference in function orientation of the two kinds of images, especially the corresponding relationship between the fluorescence area in GFP images and the protein distribution in cells, the fluorescence area is firstly extracted from the original two images, then the VIF between fused image and phase contrast image is calculate, and thirdly the VIF between fused image and fluorescence image is calculated too. Fused image should keep high similarity with both phase contrast image and fluorescence image in fluorescence area. However, in the other area, only the similarity between it and the phase contrast image is considered. Therefore, this paper first segments both fused image and source image into fluorescence area and nonfluorescence area with Otsu method [20] and calculates VIF between fused image and source image in fluorescence area and nonfluorescence area, respectively. The calculation procedure is shown in Figure 10.  Table 1 displays the calculation result of VIF of the fused image in Figure 9.

In the table, superscript fl refers to the fluorescent area while nfl refers to nonfluorescence area, *A* represents GFP fluorescence image, and *B* represents phase contrast image. VIF^*A*-fl^ refers to the similarity between fused image and GFP fluorescence image in fluorescence area, and VIF^*B*-fl^ refers to the similarity between fused image and phase contrast image in fluorescence area, while VIF^*B*-nfl^ refers to the similarity between fused image and phase contrast image in nonfluorescence area.

From Table 1, VIF^*B*-fl^ and VIF^*B*-nfl^ of the other three fusion methods are almost the same except T-HIS; the similar results indicate that all the detailed information of fused image comes from the phase contrast image no matter in fluorescent area or nonfluorescent area. Compared with other three methods, the proposed one we use in this experiment gets the highest VIF^*B*-nfl^; it does coincide with the observed results that the black background of the GFP image gets repressed. With luminance improved, the structural information will be well embedded in the fused image, which contributes the increase of VIF^*B*-nfl^. The method we use can still get higher VIF^*A*-fl^ and VIF^*B*-fl^, which indicates that the function information in GFP image and phase contrast image is well preserved in fluorescent area, and also the highest VIF^*B*-fl^ explains that SFL-CT can capture the structural information of the phase contrast image effectively.

**Figure 11:**
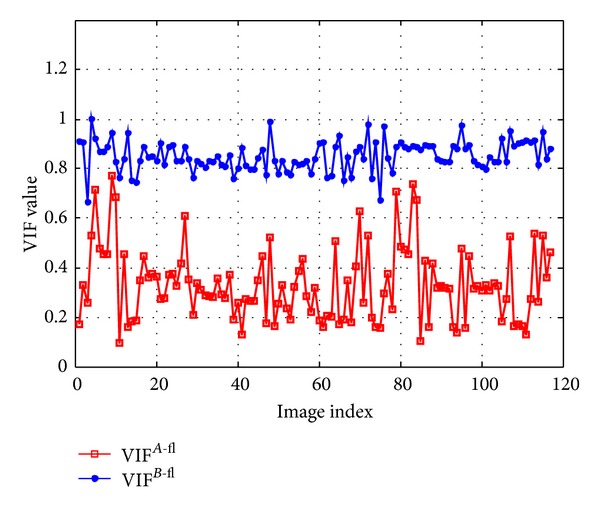
117 groups of VIF distribution histogram of Arabidopsis cell fusion image.

VIF distribution histogram of 117 groups of Arabidopsis cell fusion image is shown in Figure 11; the red squared line represents the VIF^*A*-fl^, and the blue dotted line represents the VIF^*B*-fl^. It is obvious that VIF^*B*-fl^ is higher than VIF^*A*-fl^, which does coincide with the objective of using SFL-CT to outstand the inner structural information of the phase contrast image. With the increasing VIF within fluorescent area, the VIF in nonfluorescent area also tends to improve; this indicates the following: if the intensity in fluorescent area is strengthening, VIF will increase with the function information fully reflected; and once the brightness increases, the high resolution structural information of the image can be fully shown, and the corresponding VIF^*B*-fl^ will increase; the phase image is affected by the intensity whereas low in fluorescence area, structural information cannot be reflected very well which reduces VIF's numerical similarity.

## 5. Conclusions

This paper proposes a hybrid multiscale and multilevel image fusion method based on IHS transform and SFL-CT to balance the gray structural information and molecular distribution information for the fusion of GFP image and phase contrast image. In manner of SFL-CT's advantage of directional and excellent detailed expression ability, we use SFL-CT to decompose the intensity components of both GFP image and phase contrast image, and different fusion rules are utilized for coefficients of different subbands in order to keep the localization information in GFP image and detailed high-resolution information in phase contrast image. Visual information fidelity (VIF) is introduced to assess the fusion result objectively which quantifies the similarity inside and outside the fluorescent area between the fused image and original images. The experiment fusion results of 117 groups of Arabidopsis cell images from John Innes Center demonstrate that the new algorithm can both make the details of original images well preserved and improve the visibility of the fusion image and also show the superiority of the novel method to traditional methods. Although the results of the proposed method and NSCT + PCNN look similar, the former is much better in line with the image of fused image similarity degree which means that this algorithm has made full use of the advantages of SFL-CT to keep the structural information of the phase contrast image effectively. The complexity of the algorithm is obviously lower than the latter and more advantageous to the actual application.

It is also needed to point out that from the experiment we find that VIF^*B*-fl^ is no longer equal with VIF^*B*-nfl^ when we try to improve the intensity of the fluorescent image to make a new fusion image reconstruction; this is partially due to the nonlinear relationship between similarity and intensity within fluorescent area and nonfluorescence area of the fused image. Otsu segmentation method can also cause certain disturbance to the calculation of VIF. One evaluation method cannot be perfect for different kinds of images, and a suitable fusion and evaluation method for biological cells is still a further problem to be solved.

## Figures and Tables

**Table 1:** VIF computing result.

Fusion methods	VIF^*A*-fl^	VIF^*B*-fl^	VIF^*B*-nfl^
T-IHS	0.4368	0.2759	0.4975
IHS + MRE and MAV	0.3119	0.8318	0.8318
NSCT + PCNN	0.3188	0.8992	0.8992
Hybrid NCM	0.3112	0.9299	0.9299
